# Successful removal of a deeply embedded esophageal foreign body using endoscopic submucosal dissection: a case report

**DOI:** 10.1055/a-2873-9106

**Published:** 2026-06-15

**Authors:** Haochen Zhou, Bixing Ye, Chunhua Jiao, Yixue Qin, Qi Yang, Wanyu Zhang, Guoxin Zhang

**Affiliations:** 1 Department of Gastroenterology 74734 Jiangsu Province Peopleʼs Hospital and Nanjing Medical University First Affiliated HospitalNanjingChina

**Fig. 1:**
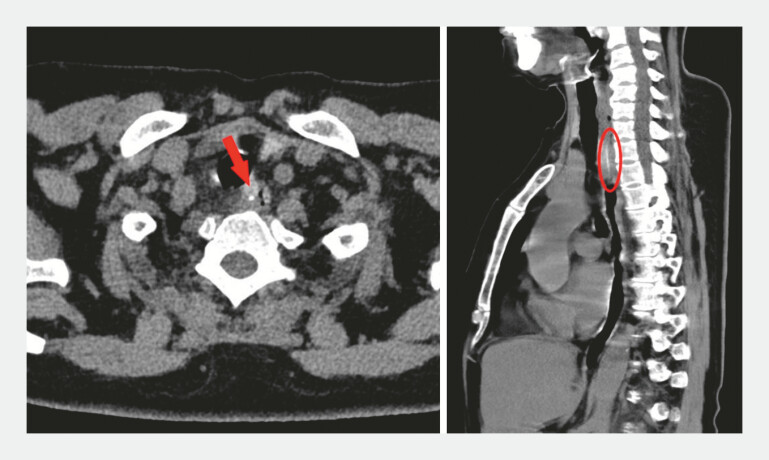
A preoperative computed tomography (CT) scan of the chest.

**Fig. 2:**
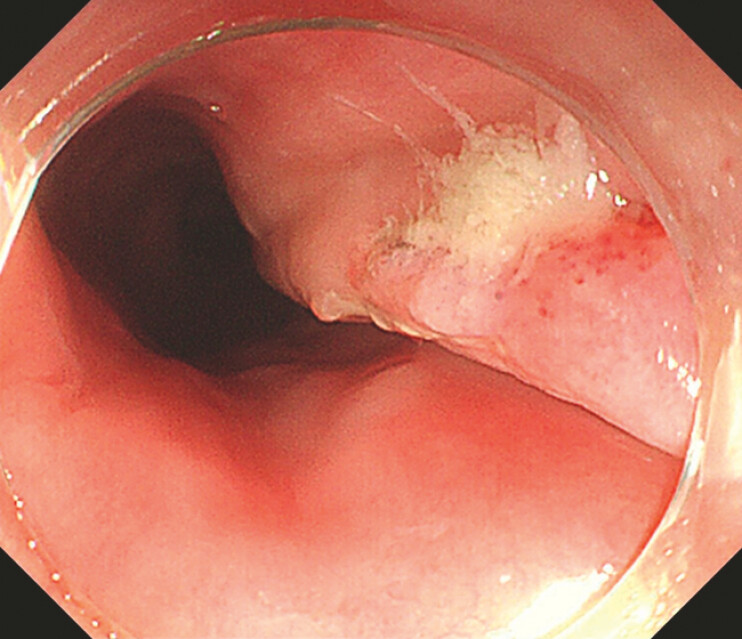
At a depth of 15–18 cm from the incisors, localized mucosal swelling and ulceration were observed.

**Fig. 3:**
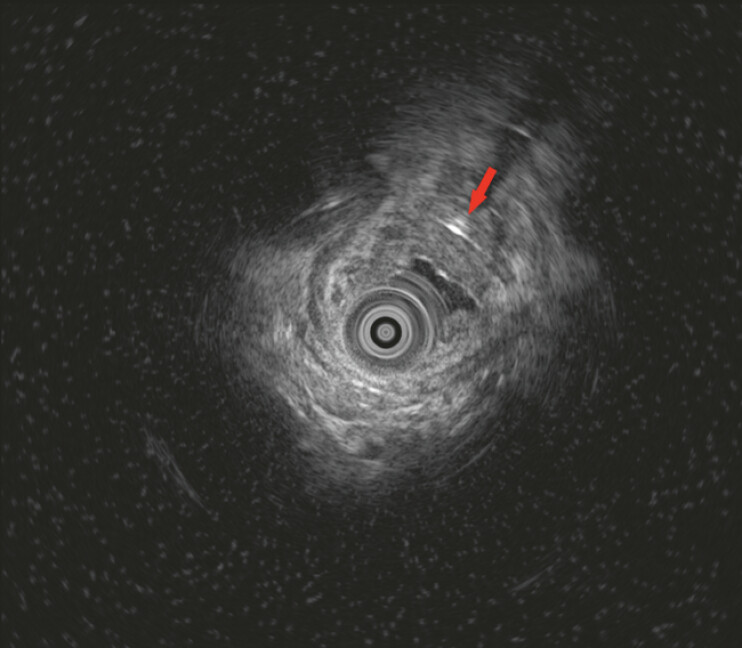
Endoscopic ultrasound (EUS) revealed punctate and linear hyperechoic foci.

A 63-year-old woman presented 4 days after accidentally ingesting a fish bone. Chest computed tomography (CT) revealed a 29-mm linear hyperdense foreign body embedded vertically in the esophageal wall (C7-T2; Fig. 1 ). Endoscopy showed edematous, ulcerated mucosa 15–18 cm from the incisors, but no visible luminal foreign body ( Fig. 2 ). Subsequent endoscopic ultrasound (EUS) demonstrated focal mucosal and submucosal thickening at the ulcer, with linear and punctate hyperechoic signals ( Fig. 3 ).

**Fig. 4:**
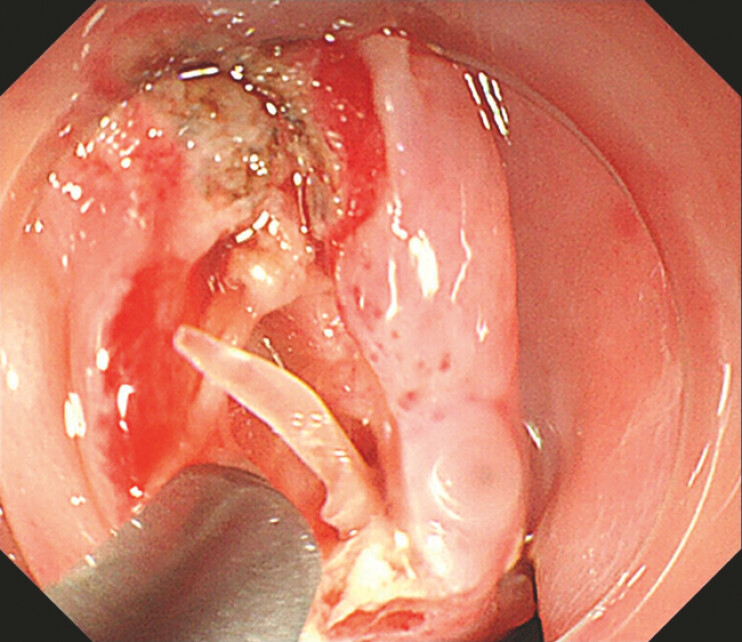
After careful dissection, the foreign body was retrieved under direct endoscopic vision using foreign body forceps.

**Fig. 5:**
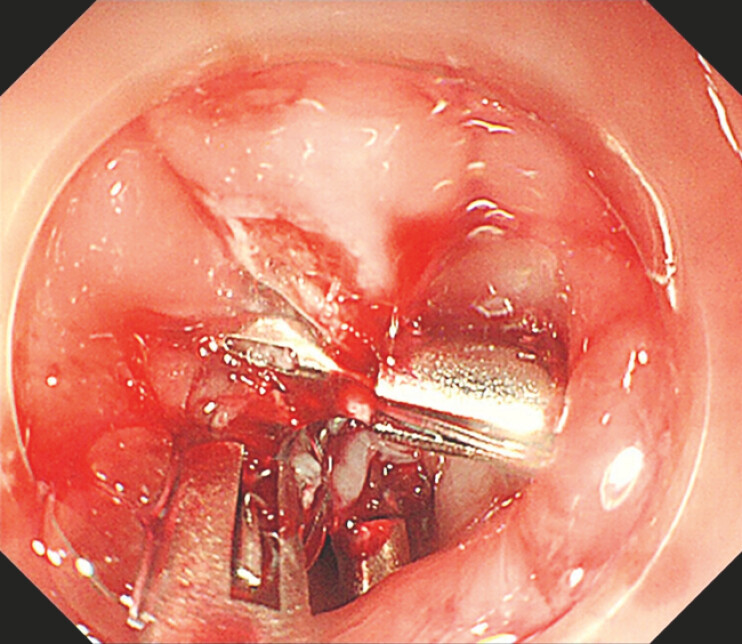
The mucosal defect was closed with six hemostatic clips.

Using a disposable high-frequency Gold Knife (Micro-Tech [Nanjing] Co., Ltd), a longitudinal mucosal incision was made at the ulcer site, revealing minimal purulent exudate. After careful dissection, an elongated fish bone was identified embedded within the submucosal layer. The foreign body was retrieved under direct endoscopic vision using foreign body forceps ( Fig. 4 ). After irrigation and hemostasis with hot forceps, follow-up EUS confirmed foreign body clearance. The defect was closed with six hemostatic clips ( Fig. 5 ). Topical norepinephrine solution controlled the bleeding, and a nasogastric tube was placed endoscopically. The foreign body measured 2.5 cm ( Video 1 ).

Modified endoscopic submucosal dissection for the successful extraction of a deeply embedded esophageal fish bone following precise EUS localization. EUS, endoscopic ultrasound.Video 1

A 1-week CT showed no complications or residual foreign body. The nasogastric tube was removed, and the patient tolerated a normal diet asymptomatically at the 1-month follow-up.

Foreign body impaction in the upper gastrointestinal tract can lead to severe complications 1 . Clinical guidelines emphasize the necessity of urgent endoscopic intervention 2 . While endoscopic submucosal dissection (ESD) is viable for extracting deeply embedded foreign bodies 3 , this case highlights the successful extraction of an initially occult esophageal object using a modified ESD approach following precise CT and EUS localization. Specifically, we intentionally omitted the submucosal injection of indigo carmine. This prevents dye-induced visual obscuration, providing a clearer endoscopic field that facilitates faster, more accurate localization and retrieval. When conventional grasping proves insufficient, this refined ESD technique serves as a highly effective salvage strategy, averting invasive surgery and ensuring favorable clinical outcomes.

Endoscopy_UCTN_Code_TTT_1AO_2AL
